# Serious Adverse Drug Reactions Associated with Off-Label and Unlicensed Drug Use in Children: A 14-Year Analysis of VigiBase Reports from Serbia

**DOI:** 10.3390/children13040536

**Published:** 2026-04-12

**Authors:** Jovana Joksimovic, Dusan Mihajlo Spasic, Marija Kukuric, Milica Bajcetic

**Affiliations:** 1 Human Medicines Centre, Medicines and Medical Devices Agency of Serbia (ALIMS), 11221 Belgrade, Serbia; jovana.joksimovic@rhei.life; 2 Department of Pharmacology, Clinical Pharmacology and Toxicology, Faculty of Medicine, University of Belgrade, 11000 Belgrade, Serbia; dusan.spasic.dr@med.bg.ac.rs; 3 Department of Emergency Radiology, University Clinical Center of Serbia, 11000 Belgrade, Serbia; marija.kukuric@kcs.ac.rs

**Keywords:** adverse drug reactions, VigiBase, off-label use, pediatric, unlicensed drug

## Abstract

**Highlights:**

**What are the main findings?**

- Off-label and unlicensed suspected drugs in Serbian pediatric VigiBase reports were associated with higher proportions of serious adverse drug reactions than on-label or licensed drugs.
- Neonates had the highest adjusted odds of serious reports, and disproportionality analysis identified several pediatric drug–event pairs consistent with known safety concerns.

**What is the implication of the main finding?**

- Off-label and unlicensed pediatric drug use should be accompanied by closer safety monitoring, particularly in the neonatal period.
- National pharmacovigilance data can help identify priorities for safer pediatric prescribing and targeted risk-minimization measures.

**Abstract:**

**Background/Objectives**: Adverse drug reactions are associated with off-label (OL) and unlicensed (UL) drug use in children. **Methods**: We conducted a retrospective observational pharmacovigilance study based on individual case safety reports (ICSRs) and adverse drug reactions (ADRs) from VigiBase for patients aged 0–17 years to evaluate reporting patterns associated with off-label and unlicensed suspected drugs reported in Serbia. **Results**: Over 14 years, 2037 pediatric ICSRs comprising 4257 ADRs were reported. Infants (28 days–23 months) accounted for just over half of ICSRs, and systemic anti-infectives and vaccines dominated suspected drugs. Overall, 9.7% of ADRs were serious, including 12 fatal outcomes, with serious ADRs reported more often for non-vaccine than vaccine suspected drugs. Off-label and unlicensed suspected drug records accounted for 10.8% and 1.9% of suspected drug records, respectively, and had higher proportions of serious ADRs than on-label or licensed use (off-label 12.1% vs. on-label 9.4%, *p* = 0.04; unlicensed 22.9% vs. licensed 9.4%, *p* < 0.001). In the adjusted ICSR-level logistic regression, neonatal age was the strongest independent predictor of seriousness, with approximately threefold higher odds of a serious case compared with children (aOR 3.04, 95% CI 1.55–5.98). **Conclusions**: Although off-label and unlicensed drugs accounted for a minority of pediatric reports, they were associated with a higher proportion of serious reported ADRs, particularly in the neonatal period. These findings should be interpreted as pharmacovigilance reporting patterns rather than incidence-based estimates or proof of causality.

## 1. Introduction

Pediatric pharmacovigilance has become a central element of contemporary medicine, bridging the historical gap between the lack of pediatric clinical trials and the safe, evidence-based use of drugs in children [1,2]. Age-related differences in pharmacokinetics, pharmacodynamics, and pharmacogenetics can alter efficacy and increase susceptibility to adverse drug reactions (ADRs) compared with adults [1,3,4]. Recent WHO and EMA initiatives stress that children should not be treated as “therapeutic orphans,” yet implementation remains inconsistent worldwide [5]. Persistent gaps in drug development, formulation, and labeling mean children remain among the most therapeutically neglected populations [4,6]. As a consequence, off-label and unlicensed prescribing remains common in pediatric care. OL use refers to administering a licensed drug outside its approved summary of product characteristics, while UL use involves drugs without marketing authorization or drugs prepared extemporaneously in hospital pharmacies [7,8]. Although these practices can address therapeutic gaps, they may proceed without robust pediatric safety evaluation, especially in neonates and infants [4,6].

Off-label and unlicensed prescribing is documented worldwide across inpatient and outpatient settings, with high rates reported in contemporary syntheses and region-specific studies in Europe, Asia, and other regions [9,10,11,12,13]. Neonatal and pediatric intensive care units frequently show the highest exposure [14].

Therapeutic domains with the greatest burden are consistent across settings. Anti-infectives are the most frequent off-label and unlicensed therapies, often reflecting formulation gaps and age-inappropriate dosing. Psychotropics, cardiovascular agents, and respiratory drugs follow, with very high off-label rates reported in child and adolescent psychiatry for non-ADHD indications [10,11,12,15].

In contrast to the well documented prevalence of off-label and unlicensed drug use, fewer prospective ward-based investigations and focused ADR reviews have examined its association with ADR outcomes. Available evidence generally reports higher odds or incidence of ADRs compared with labeled use, although absolute risk varies by drug class, age, and comorbidity [16,17,18]. Antibiotics dominate pediatric off-label and unlicensed use and have a central place in ADR concerns and the global antimicrobial resistance emergency recognized by the United Nations [12,19]. Pharmacovigilance databases are increasingly used to identify signals of inappropriate antibiotic use and antimicrobial-resistance-related harms, linking pharmacovigilance to stewardship at scale [20,21].

Despite regulatory advances and growing awareness of pediatric drug safety, off-label and unlicensed prescribing remains common because pediatric evidence, formulations, and age-appropriate dosing guidance remain incomplete. This creates a persistent tension between therapeutic necessity and safety uncertainty, particularly in neonates and infants. In this context, pharmacovigilance databases can provide valuable real-world insight into reporting patterns associated with off-label and unlicensed use and help identify priorities for safer pediatric prescribing. Against this background, the present study examined Serbian pediatric VigiBase reports over a 14-year period.

Within this global context, Serbian pediatric pharmacotherapy faces similar challenges in an evolving regulatory landscape. The national pharmacovigilance authority participates in the WHO Program for International Drug Monitoring, marketing authorization holders submit periodic safety updates, and healthcare professionals and patients contribute spontaneous reports. All children up to 18 years have free mandatory health insurance coverage, and regulatory amendments allow National Health Insurance Fund reimbursement of selected off-label uses in hospitals and clinics when an institutional ethics committee confirms clinical necessity and informed consent is obtained [22]. Nevertheless, formulary analyses indicate incomplete authorization status for pediatric drugs [23], and a cardiology ward study reported that nearly half of the prescriptions were off-label and one in ten unlicensed, underscoring continued reliance on these practices in specialized care [24].

However, national pharmacovigilance analyses in Serbia that explicitly link off-label and unlicensed exposure with pediatric ADR reporting remain limited. To address this gap, we analyzed 14 years of national pharmacovigilance data for Serbian children aged 17 years and younger to characterize ADR reporting patterns in relation to off-label and unlicensed drug use. Because VigiBase is an ICSR database, this study was designed to describe reporting patterns and explore signals of disproportionate reporting rather than estimate incidence or establish causal risk.

## 2. Materials and Methods

This retrospective pharmacovigilance study assessed suspected ADRs in Serbian children (0–17 years) with categorization by licensing status (licensed, off-label, unlicensed). The primary objective was to compare the proportion of serious adverse reactions across licensing categories; the secondary objectives were to describe age and therapeutic distributions and identify disproportionality signals.

Data were sourced from VigiBase, the WHO global database of individual case safety reports (ICSR), maintained by the Uppsala Monitoring Center under the WHO Program for International Drug Monitoring [25]. The Serbian national pharmacovigilance center within Medicines and Medical Devices Agency of Serbia (ALIMS) provided an anonymized extract of pediatric reports received between 1 January 2008 and 31 December 2021, compiled from spontaneous notifications and periodic safety updates submitted by marketing authorization holders. Causality was assessed with the WHO-UMC system [26,27].

Each ICSR represents one case and may include multiple suspected drugs and multiple adverse reactions according to EMA’s GVP Module VI. Age groups followed European Medicines Agency guidance: newborns 0–27 days, infants 28 days-23 months, children 2–11 years, adolescents 12–17 years [28]. Records with unknown age were retained in overall counts and excluded from age-stratified analyses. Products were grouped as vaccines versus drugs. Drugs were coded to the Anatomical Therapeutic (ATC) Chemical system [29]. Adverse reactions were encoded using MedDRA Preferred Term and System Organ Class. Seriousness was taken as recorded in the ICSR, and medically important events were identified using the EMA Important Medical Events list [30].

Licensing status was determined against national sources, including the ALIMS online register of authorized drugs, the Summary of Product Characteristics, and the National Medicine Registry [31].

Categories were defined as: licensed (within SmPC), off-label(outside authorized indication, age, dose, contraindication or route), and unlicensed (not authorized for pediatric use by ALIMS or lacking national marketing authorization). For off-label prescriptions, the type of deviation from the SmPC was recorded.

The primary outcome was the proportion of serious ADRs by licensing status. Annual ICSR trends were evaluated using ordinary least-squares linear regression. Disproportionality analysis was performed at the report level for pre-specified selected drug–ADR pairs chosen from the most frequently reported ADRs and their most commonly co-reported suspected drugs. For each pair, reporting odds ratios (RORs) and 95% confidence intervals were calculated using a standard case/non-case approach within the pediatric Serbian VigiBase extract. A signal of disproportionate reporting was defined as an ROR > 1 with the lower bound of the 95% confidence interval > 1. These analyses were exploratory and intended for signal detection rather than causal inference or direct comparison of drug safety profiles. For each drug–event pair, disproportionality was evaluated relative to all other reports in the study dataset not containing that specific combination.

Statistical analyses were performed using custom scripts written in Python 3.14.0 and executed in Visual Studio Code version 1.116, using the pandas 2.3.3, NumPy 2.3.5, SciPy 1.16.3, and statsmodels 0.14.5 libraries.

Data were fully anonymized by ALIMS prior to transfer; no individual identifiers were available to investigators. Formal ethics committee review was not required for secondary analysis of anonymized pharmacovigilance data [25].

## 3. Results

### 3.1. Demographic Characteristics and Annual Trends in Pediatric ICSRs

**Figure 1:**
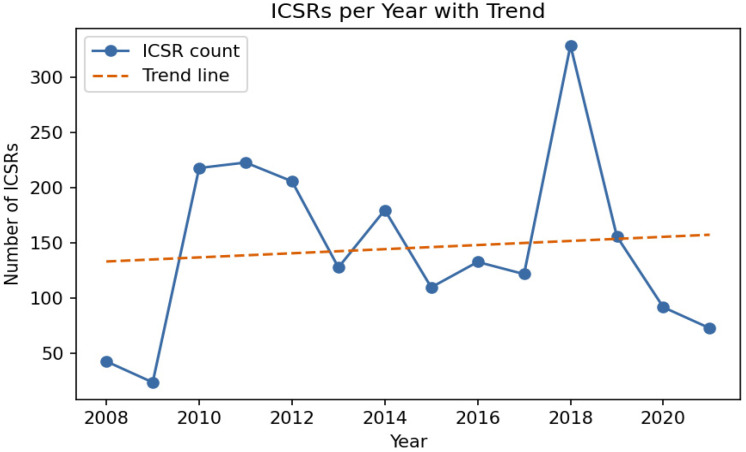
Annual number of pediatric ICSRs reported in Serbia from 2008 to 2021, with fitted linear trend line showing no significant change over time.

Between 1 January 2008 and 31 December 2021, 2037 pediatric individual case safety reports (ICSRs) were submitted. Sex distribution was 1089 males (53.5%), 902 females (44.3%), and 46 unknown (2.3%). Age distribution was 1102 infants (28 days-23 months; 54.1%), 688 children (2–11 years; 33.8%), 205 adolescents (12–17 years; 10.1%), and 39 neonates (0–27 days; 1.9%); age was unknown in three reports (0.1%). Annual reporting ranged from 24 to 329 ICSRs, with peaks in 2010–2011 and 2018, followed by a decline from 2019 onwards; linear regression showed no significant secular trend over the study period (*p* = 0.744) (Figure 1).

#### Reporter Characteristics

Physicians submitted most pediatric ICSRs (74.5%). In serious reports, other medical personnel contributed a larger share than in non-serious reports (20.7% vs. 9.7%), while physician-submitted reports comprised a smaller share of serious than non-serious cases (68.5% vs. 75.6%) (Table 1).

### 3.2. Distribution of Suspected Drugs by ATC Class

**Figure 2:**
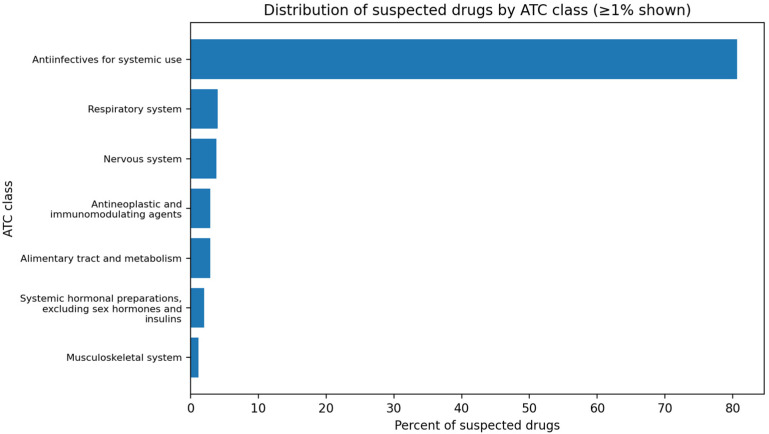
Distribution of suspected drugs by ATC class in pediatric ICSRs, 2008–2021.

Figure 2 summarizes suspected drugs by ATC class. Across 2037 ICSRs, 2354 suspected drugs were recorded. Anti-infectives for systemic use predominated (*n* = 1897; 80.6%). Smaller contributions were observed for respiratory system drugs (*n* = 94; 4.0%), nervous system drugs (*n* = 89; 3.8%), alimentary tract and metabolism drugs (*n* = 67; 2.9%), and antineoplastic and immunomodulating agents (*n* = 67; 2.9%). Systemic hormonal preparations excluding sex hormones and insulins accounted for 2.0% (*n* = 48), and musculoskeletal drugs for 1.2% (*n* = 27). All remaining ATC groups each contributed under 1%, and no antiparasitic agents were recorded.

### 3.3. Licensing and Labeling Status of Suspected Drugs

**Figure 3:**
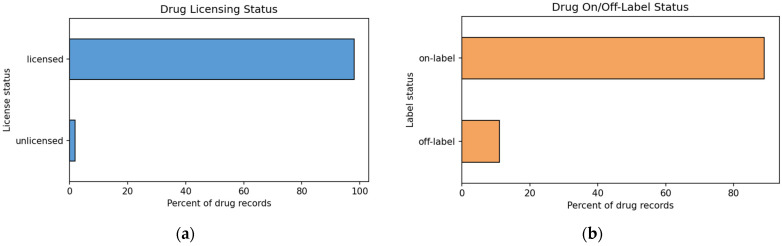
(**a**) Distribution of licensed and unlicensed suspected drugs in pediatric ICSRs, 2008–2021; (**b**) Distribution of on-label and off-label use among suspected drugs in pediatric ICSRs, 2008–2021.

From 2008 to 2021, most suspected drugs were licensed. Overall, 97.8% of suspected drug records were licensed and 1.9% unlicensed (Figure 3a). Off-label use accounted for 10.8% of suspected drug records, while 87.4% were on-label (Figure 3b).

Unlicensed drug records remained uncommon across age groups, with the highest proportion in adolescents (7.5%), followed by children (2.4%), neonates (2.3%), and infants (0.6%). Off-label use showed a stronger age gradient, reaching 39.5% in neonates, 16.8% in children, 13.2% in adolescents, and 6.2% in infants.

Among off-label entries, most involved use in a non-recommended age group (60.0%), followed by non-authorized indication (20.8%), non-recommended route (8.6%), higher-than-recommended dose (8.6%), and lower-than-recommended dose (2.0%).

#### ATC Distribution of Off-Label and Unlicensed Suspected Drugs

ATC class distributions differed between off-label and unlicensed suspected drugs (Table 2). Off-label suspected drugs were mainly anti-infectives for systemic use (65.10%), followed by respiratory system drugs (10.98%) and nervous system drugs (6.27%). Antineoplastic and immunomodulating agents (5.49%) and musculoskeletal system drugs (3.53%) contributed smaller shares, and all remaining ATC groups each accounted for 2.35% or less.

Unlicensed suspected drugs were dominated by antineoplastic and immunomodulating agents (51.11%) and anti-infectives for systemic use (24.44%). Respiratory system drugs (8.89%) and cardiovascular drugs (4.44%) were less frequent, and all other ATC groups each accounted for 2.22% or less, including sensory organ drugs (2.22%).

### 3.4. Burden and Seriousness of Adverse Drug Reaction

**Figure 4:**
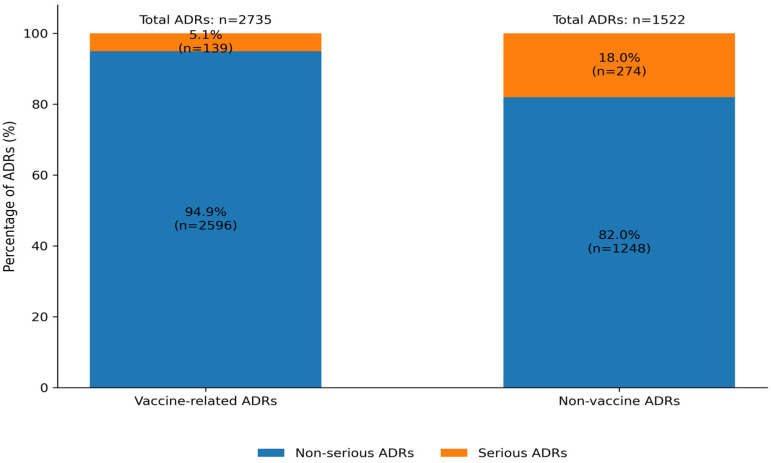
Distribution of total and serious ADRs according to vaccine and non-vaccine status.

Across all pediatric ICSRs, 4257 adverse drug reactions (ADRs) were identified after deduplicating repeated ADR terms within the same report. Of these, 413 ADRs (9.7%) were classified as serious. Vaccines accounted for 2735 ADRs, including 139 serious events (5.1% of vaccine-related ADRs), while non-vaccine drugs were associated with 1522 ADRs, of which 274 (18.0%) were serious (Figure 4).

**Figure 5:**
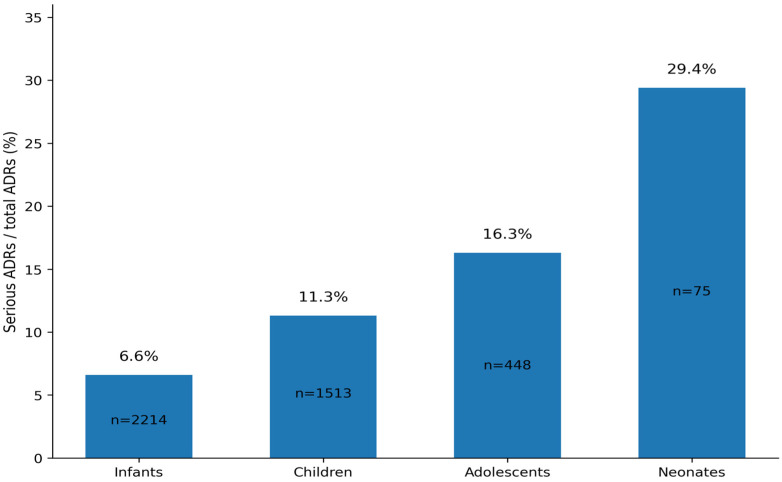
Number of total ADRs and percentage of serious ADRs by pediatric age group.

ADRs were most frequently reported in infants (2214; 52.0%), followed by children (1513; 35.5%), adolescents (448; 10.5%), and neonates (75; 1.8%); seven ADRs (0.2%) occurred in reports with unknown age. The proportion classified as serious was highest in neonates (29.4%), followed by adolescents (16.3%), children (11.3%), and infants (6.6%) (Figure 5).

### 3.5. ADR Rates by Licensing and Labeling Status

**Figure 6:**
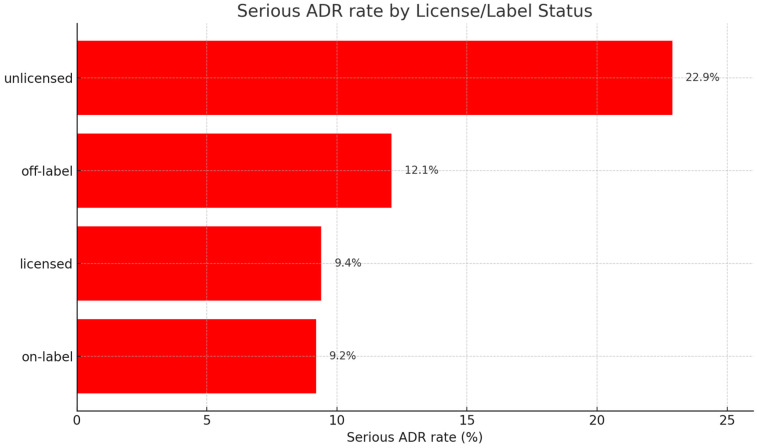
Serious ADR rates by licensing and labeling status, showing the highest proportion of serious reactions with unlicensed and off-label drugs.

When ADRs were stratified by licensing status, most were linked to licensed suspected drugs. Licensed drugs accounted for 4180 ADRs, of which 392 (9.4%) were serious. Unlicensed drugs were associated with 83 ADRs, including 19 serious reactions (22.9%). This difference in serious ADR proportions was statistically significant (χ^2^(1) = 17.1, *p* < 0.001) (Figure 6).

By labeling status, on-label use accounted for 3724 ADRs with 344 serious reactions (9.2%), while off-label use was associated with 512 ADRs including 62 serious events (12.1%). The higher proportion of serious ADRs under off-label use was statistically significant (χ^2^(1) = 4.3, *p* = 0.04). As noted in Section 3.3, off-label use was most frequent in neonates, whereas unlicensed use was proportionally most frequent in adolescents.

### 3.6. Frequently Reported ADRs

**Figure 7:**
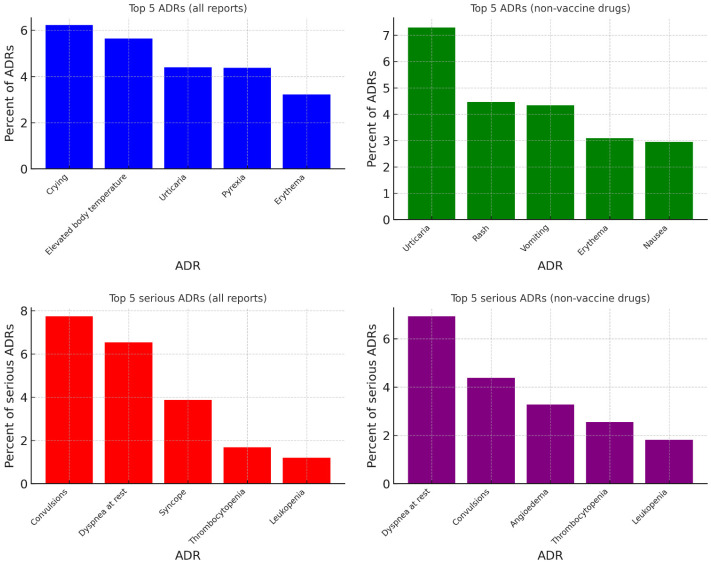
Top five most frequent ADRs and serious ADRs in pediatric ICSRs, overall and excluding vaccine-related reports.

Most frequently reported ADRs are summarized in Figure 7. Overall, the most common events were reactogenicity-type vaccine reactions such as crying and fever. After excluding vaccine-linked ADRs, cutaneous and gastrointestinal reactions predominated. Among serious ADRs, convulsions and dyspnea at rest were the most frequent, both overall and in the non-vaccine subset, while angioedema ranked among the most frequent serious events after vaccine exclusion.

### 3.7. Exploratory Disproportionality Analysis and Signals of Disproportionate Reporting for Selected Drug–ADR Pairs

**Figure 8:**
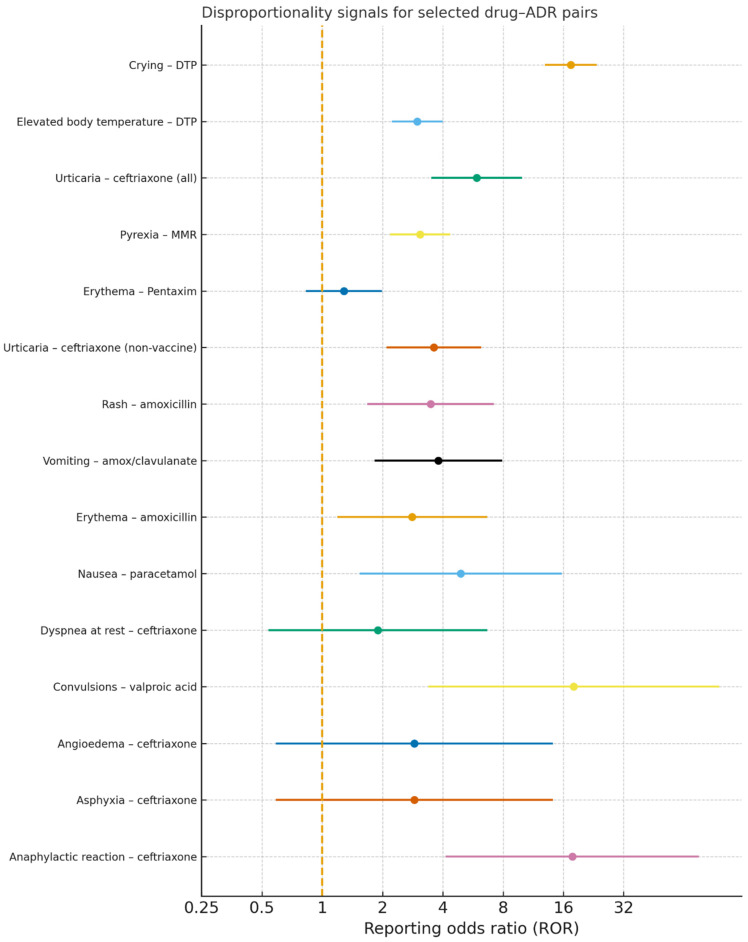
Reporting odds ratios for selected drug–ADR pairs, including common and serious reactions in all and non-vaccine pediatric reports.

To explore signals of disproportionate reporting, we calculated report-level reporting odds ratios (RORs) for selected drug–ADR pairs. (Figure 8). Crying was disproportionately reported with the DTP vaccine (ROR approximately 17.4), and elevated body temperature was also disproportionately reported with DTP (ROR approximately 3.0). Urticaria was disproportionately reported with ceftriaxone (ROR approximately 5.9), and pyrexia with the MMR vaccine (ROR approximately 3.1). The association between erythema and the pentavalent Pentaxim vaccine was modest (ROR approximately 1.3).

When restricted to non-vaccine drugs, urticaria remained disproportionately reported with ceftriaxone (ROR approximately 3.6). Rash and erythema were disproportionately reported with amoxicillin (ROR approximately 3.5 and 2.8), vomiting with amoxicillin/clavulanic acid (ROR approximately 3.8), and nausea with paracetamol (ROR approximately 4.9).

Among serious ADRs after excluding vaccine-linked reports, convulsions were disproportionately reported with valproic acid (ROR approximately 18.0) and anaphylactic reactions with ceftriaxone (ROR approximately 17.7). Ceftriaxone also showed elevated RORs for angioedema and asphyxia (both ROR approximately 2.9), while dyspnea at rest showed a smaller increase with ceftriaxone (ROR approximately 1.9).

### 3.8. Predictors of ICSR Seriousness: Multivariable Logistic Regression

To identify factors independently associated with seriousness, we performed an ICSR-level multivariable logistic regression, defining a serious case as an ICSR containing at least one serious ADR.

Neonatal cases had the highest odds of seriousness compared with children (aOR 3.04, 95% CI 1.55–5.98). Seriousness increased with a greater number of suspected drugs (per additional suspected drug: aOR 1.57, 95% CI 1.24–1.98).

Compared with physician reports, reports submitted by other medical personnel were more likely to be serious (aOR 1.73, 95% CI 1.20–2.48), while pharmacist reports were less likely to be serious (aOR 0.44, 95% CI 0.22–0.89).

Vaccine involvement was associated with lower odds of seriousness (aOR 0.33, 95% CI 0.23–0.48). Relative to anti-infectives for systemic use, seriousness was higher when the primary suspected drug belonged to the nervous system class (aOR 2.00, 95% CI 1.17–3.41) or musculoskeletal system class (aOR 2.50, 95% CI 1.06–5.87). After adjustment, off-label and unlicensed status were not independently associated with seriousness.

These adjusted odds ratios reflect predictors of reported ICSR seriousness within the submitted pharmacovigilance dataset and should not be interpreted as incidence-based risk estimates in the underlying treated pediatric population.

### 3.9. Most Frequently Reported Unlicensed and Off-Label Suspected Drugs

Among unlicensed suspected drugs, budesonide and asparaginase were the most frequently reported, with four ICSRs each, followed by cloxacillin, levofloxacin, and carboplatin with three each. Among off-label suspected drugs, Pentaxim predominated by a wide margin, followed by the BCG vaccine, montelukast, the MMR vaccine, and vancomycin.

Table 3 summarizes the unlicensed drugs most frequently reported as suspected drugs. Serious ADRs were recorded for budesonide (*n* = 1), asparaginase (*n* = 2), cloxacillin (*n* = 2), and carboplatin (*n* = 1), and none were recorded for levofloxacin. Where specified, seriousness most commonly reflected hospitalization or prolonged hospitalization; one carboplatin-associated report had a fatal outcome. The most frequently listed ADRs for these drugs are shown in Table 3.

Table 4 summarizes the off-label drugs most frequently reported as suspected drugs. Serious ADRs were recorded for Pentaxim (*n* = 8), montelukast (*n* = 1), and vancomycin (*n* = 2), while none were recorded for the BCG or the measles, mumps and rubella vaccines. The most frequently listed ADRs for these drugs are shown in Table 4.

## 4. Discussion

Over 14 years of pediatric pharmacovigilance reporting in Serbia, 2037 ICSRs comprising 4257 ADRs were recorded, with reports concentrated in infants followed by children, adolescents, and neonates. Suspected drugs were dominated by systemic anti-infectives and vaccines. Overall, 9.7% of ADRs met seriousness criteria, including 12 fatal outcomes. Serious ADRs were proportionally more frequent in non-vaccine reports than vaccine reports (18.0% vs. 5.1%), and neonates had the highest seriousness proportion despite few reports. Importantly, although off-label and unlicensed suspected drugs were associated with higher unadjusted proportions of serious ADRs, licensing status did not remain an independent predictor of seriousness in the adjusted ICSR-level model, suggesting that age, polypharmacy, vaccine status, and therapeutic class may explain a substantial part of the crude association.

The most frequently reported ADRs were crying, fever, and injection-site reactions, while convulsions and dyspnea at rest were the most common serious events. The exploratory disproportionality analyses reproduced expected vaccine reactogenicity patterns and common non-vaccine reporting profiles, and identified signals of disproportionate reporting for ceftriaxone-related hypersensitivity events that merit continued pharmacovigilance monitoring.

These patterns align with international syntheses and large European cohorts that document substantial pediatric exposure to anti-infectives and frequent treatment of respiratory and neuropsychiatric conditions in routine care, including off-label and unlicensed use across settings [9,10,11,12,13]. Prior ward-based and prospective studies often emphasized a higher incidence of adverse reactions in hospitalized children receiving off-label or unlicensed therapy [16,17,18]. A national Italian program showed that structured monitoring can capture substantial numbers of serious pediatric ADRs, including events related to off-label use [32], and expert commentary has long stressed that widespread unlicensed and off-label prescribing, particularly in neonatal and intensive care settings, likely increases toxicity risk compared with licensed use [33]. In our dataset, off-label and unlicensed exposure generated far fewer reports than licensed drugs, yet a higher unadjusted proportion of serious ADRs, consistent with developmental pharmacology and formulation constraints in early life [18].

The age gradient is consistent with evidence that formulation gaps, concentration mismatches, and non-standard routes are most acute at the extremes of age, and that immature elimination pathways and variable receptor sensitivity increase vulnerability in infants and toddlers [9,11,14,18]. Similar inpatient patterns in early life are described in Nordic cohorts [12], alongside national outpatient data from Sweden [11].

Therapeutically, anti-infectives were the largest contributors to pediatric reports, echoing observations that antibiotics are both the most prescribed and the most frequently used off-label class in children [10,12]. The WHO AWaRe framework provides a practical structure to prioritize Access to antibiotics and to constrain Watch and Reserve agents while guiding national lists and pediatric formularies [34]. Pharmacovigilance can support stewardship, as spontaneous-report analyses have been used to flag inappropriate antibiotic use and resistance-related harms for targeted investigation [20]. Aligning these approaches with the United Nations call to action on antimicrobial resistance links patient safety and public health objectives [19,21]. Similar antibiotic-led profiles are described in Nordic settings [11,12].

For nervous system and respiratory agents, the literature notes substantial off-label use and class-specific challenges, including very high off-label rates for non-ADHD indications in child and adolescent psychiatry, where dose response and tolerability may differ from adults [10,12,15]. A similar concern applies to antineoplastic and immunomodulating agents, which in our dataset included asparaginase and carboplatin among the most frequently reported unlicensed suspected drugs. This pattern is clinically plausible because children receiving anticancer therapy constitute a particularly vulnerable population, and non-label use may further increase uncertainty around age-appropriate dosing, exposure, and safety in the setting of developmental pharmacokinetic variability [35]. For carboplatin, severe toxicity or even fatal outcomes may be more likely under unlicensed or otherwise non-label use when exposure is not optimally individualized, because platinum toxicities are exposure dependent and can affect non-target tissues through DNA damage and oxidative injury; clinically important toxicity, including ototoxicity, becomes more likely at higher or more intensive exposures (e.g., renal immaturity) [36]. By contrast, the relatively high rate of hypersensitivity associated with asparaginase is well recognized even during standard on-label treatment, reflecting the intrinsic immunogenicity of this bacterial-derived enzyme; other major toxicities include pancreatitis, thrombosis, encephalopathy, and liver dysfunction [37].

Several mechanisms may contribute to higher severity in off-label and unlicensed exposure, including clustering in higher acuity care, complex comorbidity, and polypharmacy, as well asdosage form problems and risks introduced by hospital compounding; the drug mix also matters because anti-infectives and psychotropics carry predictable toxicities when indication, dose, or route diverges from labeling in young patients [10,12,15].

The regulatory context helps explain why these patterns persist. Despite the EU Paediatric Regulation and Pediatric Investigation Plans, fewer than one third of newly approved drugs arrive with complete pediatric labeling, and incentives for off-patent drugs have achieved uneven gains in child-appropriate formulations [38]. Meta-analytic evidence indicates that increased pediatric trial activity has not reliably translated into labeled indications across high-need areas, sustaining reliance on empirical dose adjustment and extemporaneous preparation in practice [9]. Uganda’s experience with vaccine pharmacovigilance illustrates how underreporting and limited analytical capacity delay early signal detection, challenges that generalize to pediatric drug safety in many low- and middle-income settings [39]. Broader guidance on pharmacovigilance in special populations emphasizes preventability through structured dosing, safer formulation practices, and targeted education [5].

Several practice-facing levers follow directly from these results: standardized age-based dosing charts, validated dilution tables, and protocols for extemporaneous compounding, alongside the wider availability of ready-to-use pediatric formulations for high-impact antibiotics. Work within the EU LENA program on enalapril orodispersible minitablets shows that age-appropriate solid formulations can be developed with acceptable pharmacokinetics, safety, and strong acceptability in infants and young children [40,41]. The Catalonian Self-Audit model indicates that feedback-informed primary care can curb inappropriate prescribing and could be adapted to pediatric contexts in Serbia [42].

Emerging methods can strengthen pediatric safety surveillance, including class-aware analytics using International Nonproprietary Name stems, integration of real-world data with spontaneous reporting, and electronic prescribing self-audit tools [39,42,43].

Machine learning over spontaneous reports and electronic records can detect shifts in severity distributions that simple counts overlook [43]. Integrated approaches combining real-world data, biomedical knowledge bases, and postmarketing surveillance can map pediatric safety knowledge gaps and prioritize verification studies [44].

A major strength of this study is the use of a 14-year national pediatric pharmacovigilance dataset, which enabled simultaneous evaluation of age patterns, suspected drug classes, licensing status, and exploratory disproportionality signals in a real-world setting. However, several limitations must be acknowledged. As a spontaneous reporting analysis, the study is subject to underreporting, variable report completeness, and absence of exposure denominators, which precludes incidence estimation. The observational pharmacovigilance design also does not permit causal inference. In addition, residual confounding by indication, age, polypharmacy, and reporting behavior cannot be excluded. Because the spontaneous reporting dataset did not provide sufficiently standardized information on the severity of the underlying treated condition, no robust analysis of off-label or unlicensed use according to disease severity was performed. Finally, reporting patterns in Serbian VigiBase-linked data may reflect local prescribing, reimbursement, product availability, and national reporting practices, which may limit generalizability.

In summary, Serbian pediatric pharmacovigilance data show that off-label and unlicensed exposure represents a minority of reports, yet it is associated with a higher proportion of serious reported ADRs in unadjusted analyses. Because these findings derive from spontaneous ICSR data, they should be interpreted as reporting patterns and signals rather than direct estimates of comparative clinical risk. Priorities are to strengthen preventability through pediatric dosing and compounding standards, align antibiotic use with AWaRe, deploy prescribing self-audit and point-of-care decision support, and adopt class-aware and data-linked pharmacovigilance analytics, while maintaining routine monitoring of high-impact signals such as ceftriaxone-associated hypersensitivity. These steps would narrow the gap between necessary off-label and unlicensed practice and safe pediatric care while advancing antimicrobial stewardship and national drug policy goals [19,20,21,34].

## Figures and Tables

**Table 1:** Reporter type distribution for pediatric ICSRs overall and stratified by seriousness (*n*, %).

Reporter Type	All Cases, *n* (%)	Serious Cases, *n* (%)	Non-Serious Cases, *n* (%)
Physician	1517 (74.5)	228 (68.5)	1289 (75.6)
Other medical personnel	234 (11.5)	69 (20.7)	165 (9.7)
Unknown	113 (5.5)	8 (2.4)	105 (6.2)
Pharmacist	90 (4.4)	11 (3.3)	79 (4.6)
Non-medical individual	76 (3.7)	15 (4.5)	61 (3.6)
Patient	7 (0.3)	2 (0.6)	5 (0.3)
Total	2037 (100.0)	333 (100.0)	1704 (100.0)

**Table 2:** ATC distribution of off-label and unlicensed suspected drugs (%, within status category).

ATC	Off-Label (%)	Unlicensed (%)
Alimentary tract and metabolism	1.96	0.00
Blood and blood-forming organs	2.35	0.00
Cardiovascular system	1.57	4.44
Dermatologicals	0.39	0.00
Genitourinary system and sex hormones	1.18	0.00
Systemic hormonal preparations, excluding sex hormones and insulins	1.18	2.22
Anti-infectives for systemic use	65.10	24.44
Antineoplastic and immunomodulating agents	5.49	51.11
Musculoskeletal system	3.53	2.22
Nervous system	6.27	2.22
Respiratory system	10.98	8.89
Sensory organs	0.00	2.22

**Table 3:** Top five unlicensed drugs reported as suspected drugs in pediatric ICSRs, with number of ICSRs, ADR-containing ICSRs, serious ADRs, distribution of seriousness criteria, and most frequent ADRs.

Unlicensed Drug	UL ICSRs, *n* ^a^	ADRs in UL ICSRs, *n* ^b^	Serious ADRs, *n* ^c^	Seriousness Criteria	Most Frequent ADRs
Budesonide	4	4	1	Unknown	Rash; Epistaxis
Asparaginase	4	4	2	Hospitalization/prolonged hospitalization	Anaphylactic reaction; Erythema
Cloxacillin	3	3	2	Hospitalization / prolonged hospitalization; Unknown	Dyspnea at rest; Rash
Levofloxacin	3	3	0	N/A	Urticaria; Language disorder
Carboplatin	3	3	1	Death	Pruritus; Myelosuppression

^a^ Number of distinct ICSRs where the unlicensed drug was suspected. ^b^ Total number of ADRs within all ICSRs where the unlicensed drug was suspected. ^c^ Number of serious ADRs out of all reported ADRs within all ICSRs where the unlicensed drug was suspected.

**Table 4:** Top five off-label drugs reported as suspected drugs in pediatric ICSRs, with number of ICSRs, ADR-containing ICSRs, serious ADRs, distribution of seriousness criteria, and most frequent ADRs.

Off-Label Drug	OL ICSRs, *n* ^a^	ADRs in OL ICSRs, *n* ^b^	Serious ADRs, *n* ^c^	Seriousness Criteria	Most Frequent ADRs
Pentaxim pentavalent vaccine (DTaP–IPV–Hib)	80	80	8	Hospitalization/prolonged hospitalization	Elevated body temperature; Crying
BCG vaccine	13	13	0	N/A	Lymphadenitis; Lymphadenopathy
Montelukast	13	12	1	Unknown	Urticaria
Measles–mumps–rubella (MMR) vaccine	9	9	0	N/A	Lymphadenopathy; Parotitis
Vancomycin	8	7	2	Unknown	Urticaria; Erythema

^a^ Number of distinct ICSRs where the off-label drug was suspected. ^b^ Total number of ADRs within all ICSRs where the off-label drug was suspected. ^c^ Number of serious ADRs out of all reported ADRs within all ICSRs where the off-label drug was suspected.

## Data Availability

The data analyzed in this study were obtained from VigiBase, the World Health Organization global database of individual case safety reports (ICSRs). Access to VigiBase is restricted to drug regulatory agencies that are members of the WHO Programme.
